# Supplementary material for: A treasure trove of 1034 actinomycete genomes
Source: Nucleic Acids Res. 2024 Jun 22;52(13):7487–503. doi: 10.1093/nar/gkae523 (PMC11260486; doi:10.1093/nar/gkae523)

Supplementary Figures for

A treasure trove of 1,034 actinomycete genomes

Tue Sparholt Jørgensen[1], Omkar Mohite[1], Eva B Sterndorff[1], Maria Alvarez-Arevalo[1], Kai Blin[1], Thomas J Booth[1], Pep Charusanti[1], David Faurdal[1], Troels Ø Hansen[1], Matin Nuhamunada[1], Anna-Sophie Mourched[1], Bernhard Ø Palsson[1,2], Tilmann Weber[1]

[1] The Novo Nordisk Foundation Center for Biosustainability, Technical University of Denmark, Søltofts Plads, building 220, 2800 Kgs. Lyngby, Denmark
[2]Department of Bioengineering, University of California, 417 Powell-Focht Bioengineering Hall, San Diego, La Jolla, CA 92093-0412, USA

Supplementary Figure 1 Coverage plots

Supplementary Figure 2 List of countries

Supplementary Figure 3 Core gene placement legend

Supplementary Figure 4 Protocluster type plots

Supplementary Figure 5 C-linker accession numbers

Supplementary Figure 1 example coverage plots

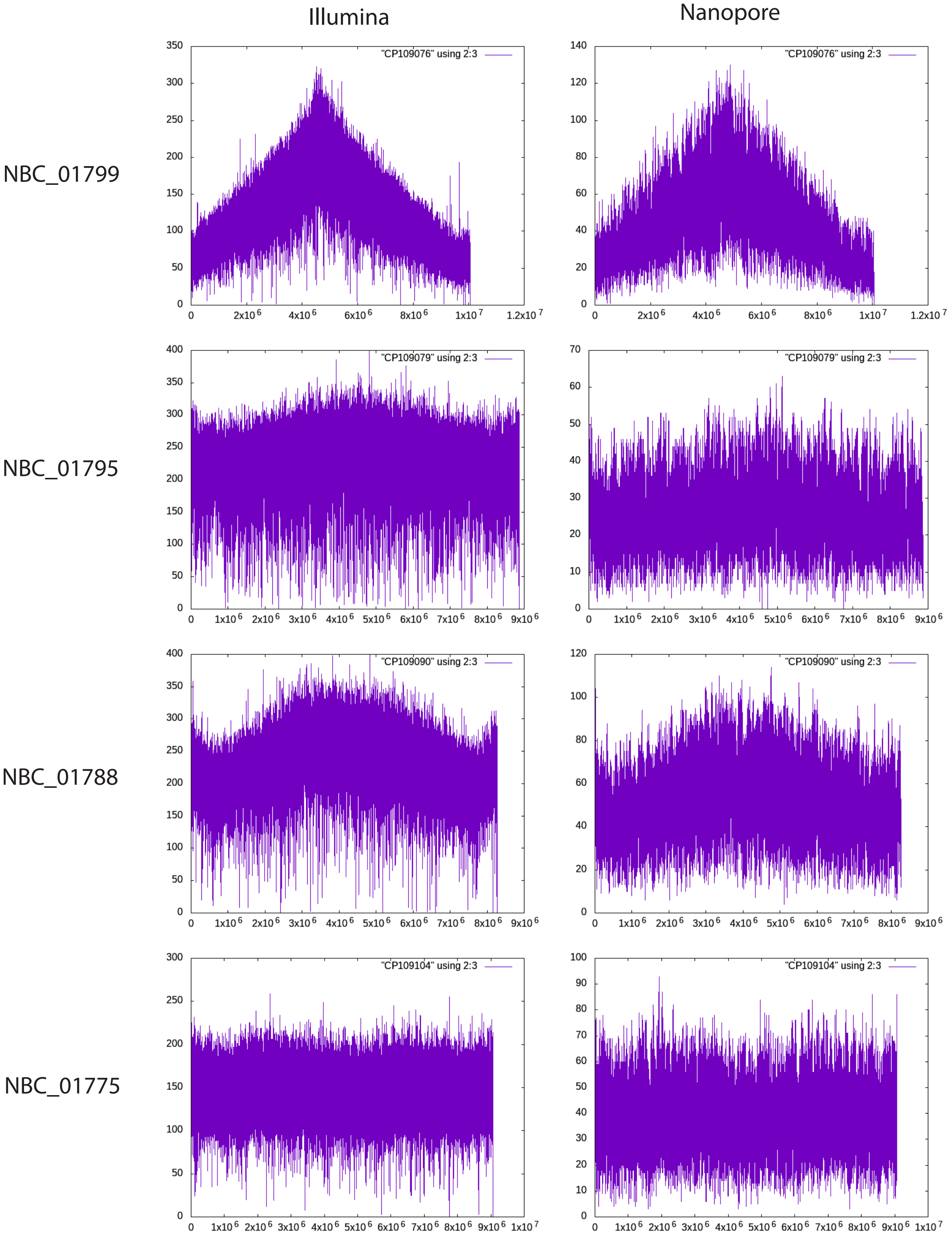

Coverage of illumina and nanopore reads on the assembled linear chromosomes of four Streptomyces strains Each of the complete chromosomes were inspected to identify sudden changes in coverage: double or half coverage could indicate the presence of large scale assembly errors. Chromosomes where such errors were observed were attempted fixed, and if Filtlong read noise reduction did not allieviate the issue, the strain was classified as 'WGS level'. The distinct 'tent' or 'moustache' coverage pattern for the strains NBC_01799 and NBC_01788 are likely due to actively growing cultures, whereas the more uniform coverage of NBC_01795 and NBC_01775 indicate stationary phase cultures.

Supplementary Figure 2 list of countries

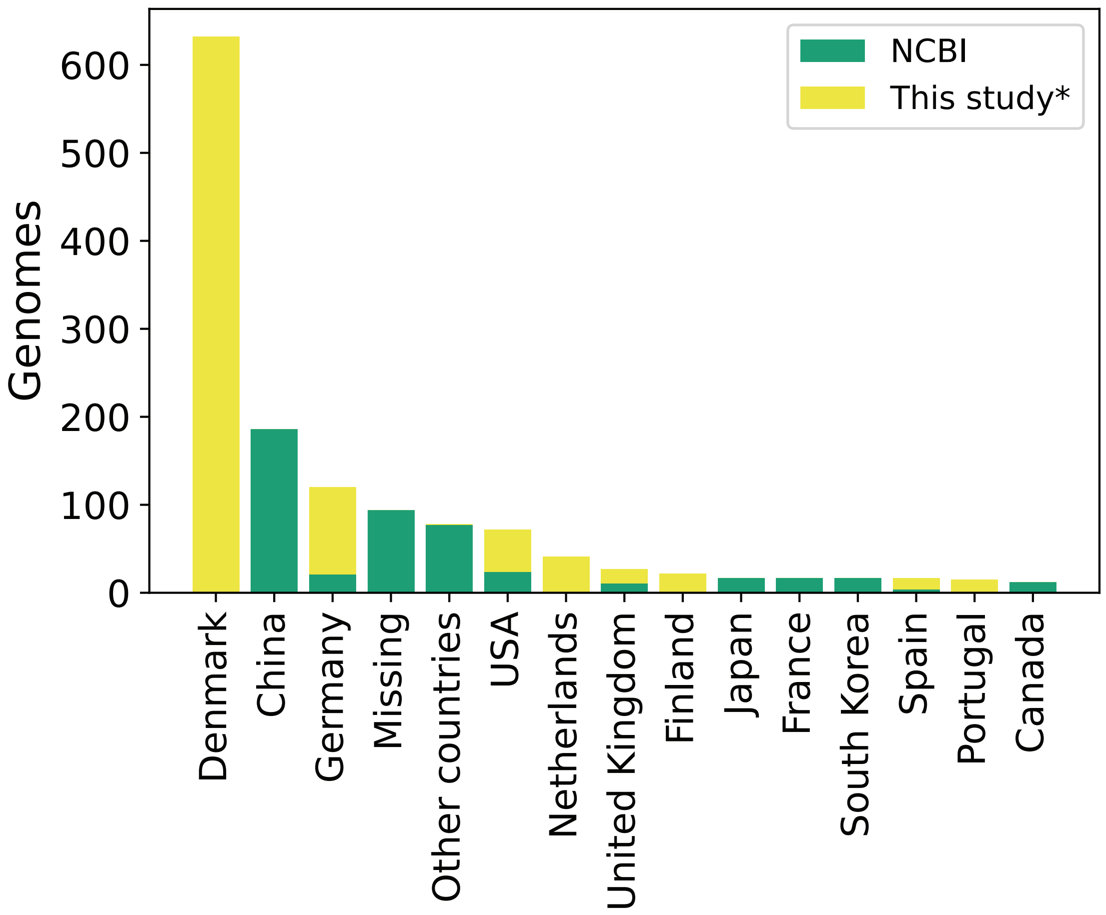

List of countries of origin, when given, for high quality genomes from this study (yellow) and from NCBI (green).

Supplementary Figure 3 legend for figure 4 core gene placement

Legend for primary core gene

- Chromosomal_replication_initiator_protein_DnaA
- Ribosomal_RNA_small_subunit_methyltransferase_G
- 30S_ribosomal_protein_S18
- 30S_ribosomal_protein_S6
- Ribosomal_protein_L9
- aspartate--tRNA_ligase
- serine--tRNA_ligase
- phosphoribosylamine--glycine_ligase
- phosphoribosylformylglycinamidine_synthase
- amidophosphoribosyltransferase
- phosphoribosylformylglycinamidine_cyclo-ligase
- adenylosuccinate_synthase
- recombination_protein_RecR
- Protein_GrpE
- DNA_topoisomerase_I
- methionine--tRNA_ligase
- DNA_repair_protein_RadA
- UDP-N-acetylenolpyruvoylglucosamine_reductase
- 50S_ribosomal_protein_L11
- 50S_ribosomal_protein_L1
- 50S_ribosomal_protein_L10
- 50S_ribosomal_protein_L7/L12
- DNA-directed_RNA_polymerase_subunit_beta
- DNA-directed_RNA_polymerase_subunit_beta'
- 30S_ribosomal_protein_S12
- 30S_ribosomal_protein_S7
- 30S_ribosomal_protein_S10
- 50S_ribosomal_protein_L3
- 50S_ribosomal_protein_L4
- 50S_ribosomal_protein_L23
- Ribosomal_protein_L2
- 30S_ribosomal_protein_S19
- 50S_ribosomal_protein_L22
- K_Homology_domain
- 50S_ribosomal_protein_L16
- 50S_ribosomal_protein_L29
- 30S_ribosomal_protein_S17
- 50S_ribosomal_protein_L14
- DNA-directed_RNA_polymerase_subunit_alpha
- 50S_ribosomal_protein_L24
- 50S_ribosomal_protein_L5
- 30S_ribosomal_protein_S8
- 50S_ribosomal_protein_L6
- Protein_translocase_subunit_SecY
- 50S_ribosomal_protein_L18
- 30S_ribosomal_protein_S5
- 50S_ribosomal_protein_L15
- tRNA_N6-adenosine_threonylcarbamoyltransferase
- adenylate_kinase
- translation_initiation_factor_IF-1
- 30S_ribosomal_protein_S13
- 30S_ribosomal_protein_S11
- 50S_ribosomal_protein_L17
- 50S_ribosomal_protein_L13
- 30S_ribosomal_protein_S9
- Peptidyl-tRNA_hydrolase
- Enolase
- tRNA_threonylcarbamoyladenosine_biosynthesis_protein_TsaE
- GMP_synthase_
- Bifunctional_purine_biosynthesis_protein_PurH
- SsrA-binding_protein
- biotin--
- GTP-binding_protein
- trigger_factor
- 50S_ribosomal_protein_L21
- 50S_ribosomal_protein_L27
- Ribosomal_silencing_factor_RsfS
- 30S_ribosomal_protein_S20
- elongation_factor_4
- ATP_synthase_subunit_delta
- ATP_synthase_subunit_alpha
- ATP_synthase_gamma_chain
- ATP_synthase_subunit_beta
- 16S_rRNA_(uracil(1498)-N(3))-methyltransferase
- Endoribonuclease_YbeY
- GTPase_Era
- DNA_repair_protein_RecO
- tRNA_2-thiouridine(34)_synthase_MnmA
- glycerol-3-phosphate_dehydrogenase
- methyltransferase
- pantetheine-phosphate_adenylyltransferase
- ribonuclease_III
- 30S_ribosomal_protein_S16
- 50S_ribosomal_protein_L19
- Ribosome_maturation_factor_RimM
- 30S_ribosomal_protein_S2
- Uridylate_kinase
- ribosome_recycling_factor
- Zinc_metalloprotease
- proline--tRNA_ligase
- Ribosome_maturation_factor_RimP
- Transcription_termination/antitermination_protein_NusA
- ribosome-binding_factor_A
- tRNA_pseudouridine_synthase_B
- Riboflavin_biosynthesis_protein
- 30S_ribosomal_protein_S15
- Protein_RecA
- tRNA_dimethylallyltransferase
- GTPase_HflX
- 16S_rRNA_(cytosine(1402)-N(4))-methyltransferase
- Phospho-N-acetylmuramoyl-pentapeptide-transferase
- UDP-N-acetylmuramoylalanine-D-glutamate_ligase
- cell_division_protein_FtsZ
- excinuclease_ABC_subunit_B
- excinuclease_ABC_subunit_C
- phosphoglycerate_kinase
- Triosephosphate_isomerase
- tyrosine--tRNA_ligase
- DNA_repair_protein_RecN
- cytidylate_kinase
- 30S_ribosomal_protein_S4
- translation_initiation_factor_IF-3
- 50S_ribosomal_protein_L35
- 50S_ribosomal_protein_L20
- phenylalanine--tRNA_ligase_subunit_alpha
- alanine--tRNA_ligase
- methionine_adenosyltransferase
- chorismate_synthase
- Holliday_junction_DNA_helicase_RuvB
- Putative_pre-16S_rRNA_nuclease
- aspartate_carbamoyltransferase
- Transcription_antitermination_protein_NusB
- guanylate_kinase
- phosphopantothenoylcysteine_decarboxylase

Legend for secondary core gene

- 30S_ribosomal_protein_S18
- serine--tRNA_ligase
- other
- Protein_GrpE
- DNA-directed_RNA_polymerase_subunit_alpha
- Protein_translocase_subunit_SecY
- tRNA_N6-adenosine_threonylcarbamoyltransferase
- adenylate_kinase
- translation_initiation_factor_IF-1
- Enolase
- glycerol-3-phosphate_dehydrogenase
- pantetheine-phosphate_adenylyltransferase
- Protein_RecA
- Phospho-N-acetylmuramoyl-pentapeptide-transferase
- 30S_ribosomal_protein_S4
- alanine--tRNA_ligase
- methionine_adenosyltransferase
- chorismate_synthase

Legend for figure 4 core gene placement. The color representing each gene is identical for the primary and duplicate core genes. The order of the genes is based on the distance to the chromosome center. The gene names are the names from the BUSCO bacteria ODB10 dataset.

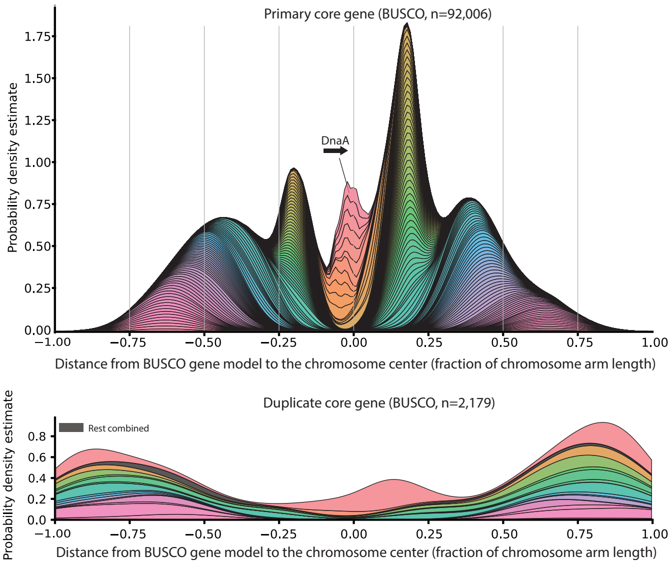

Supplementary Figure 4 protocluster types placement

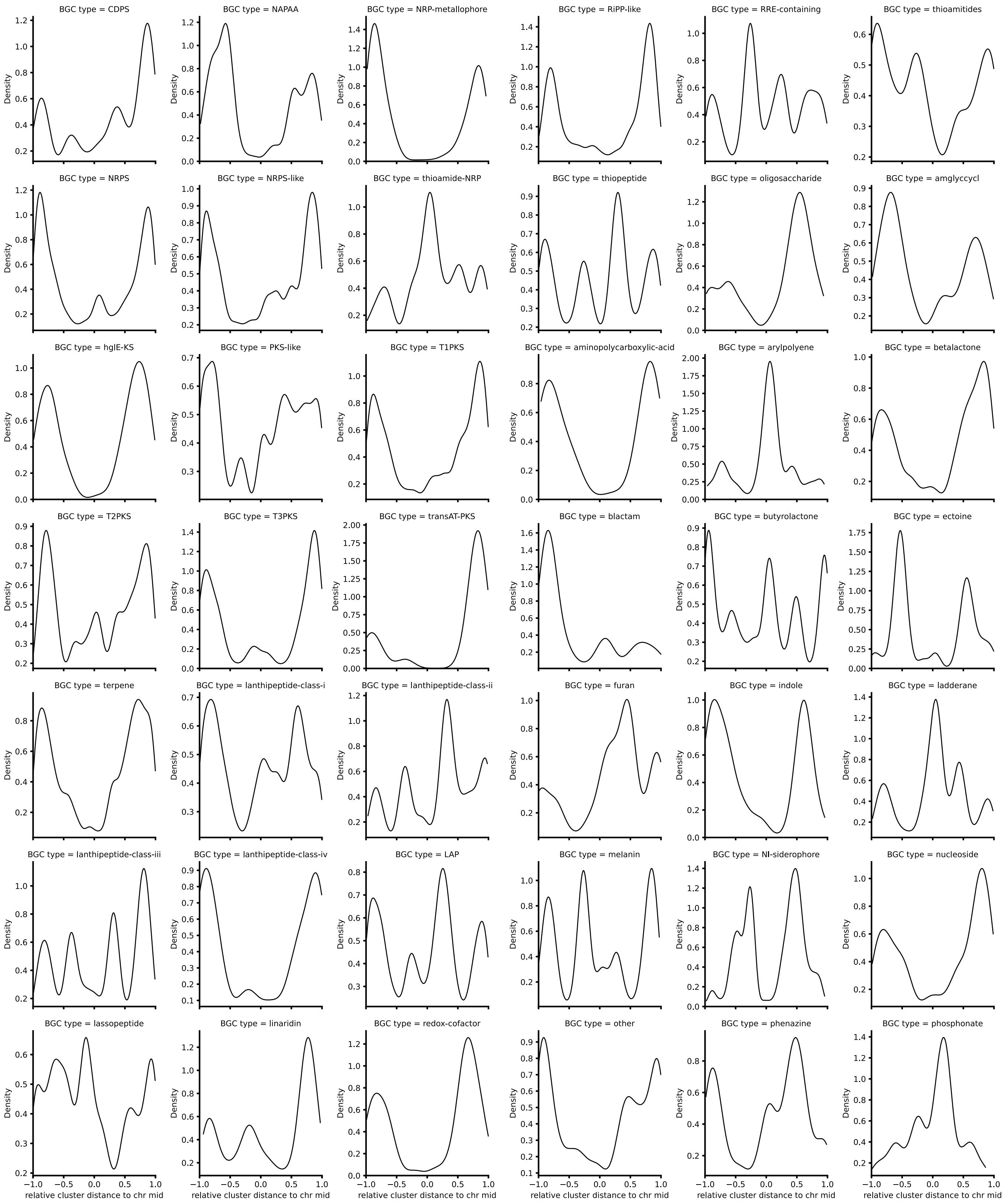

Placement of all protocluster types along the linear complete Streptomyces chromosomes. Only protocluster types with at least 50 observations are shown.

Supplementary Figure 5 accession numbers from c-linker plots

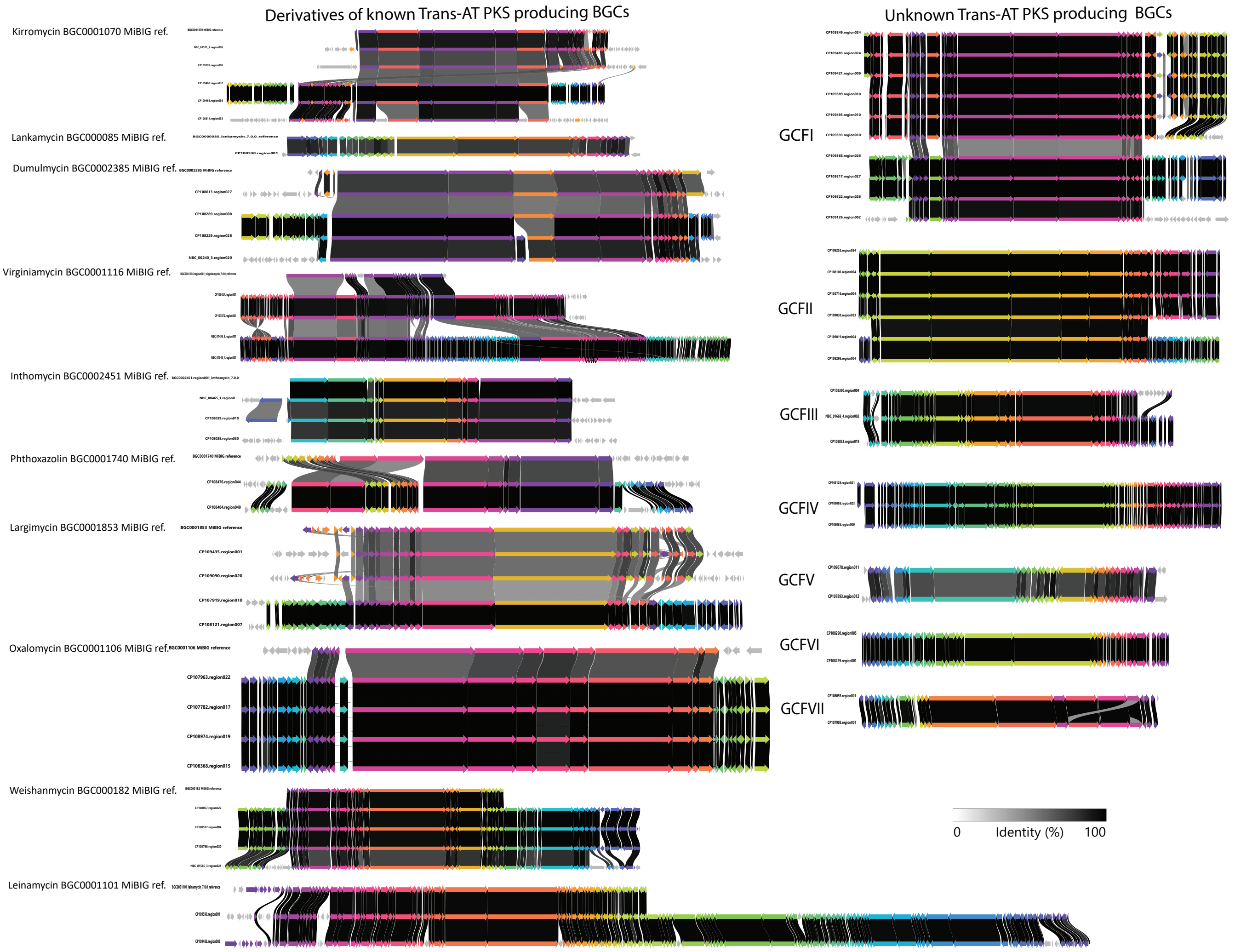

Accession numbers for the BGCs shown in Figure 6

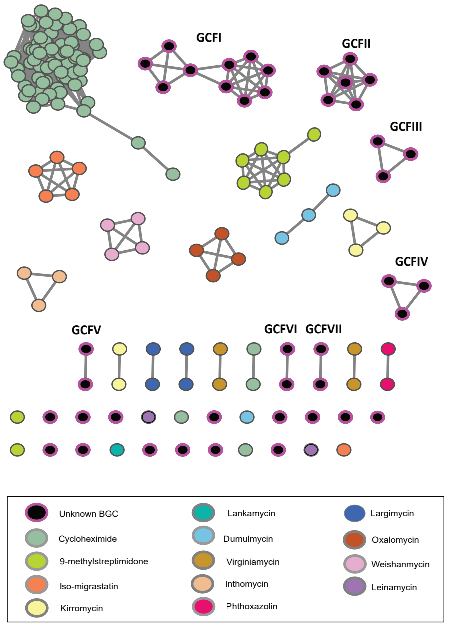

Supplement: gkae523_Supplemental_Files [file gkae523_supplemental_files.zip › Supplementary figures.pdf]
